# TRIM33 protects osteoblasts from oxidative stress‐induced apoptosis in osteoporosis by inhibiting FOXO3a ubiquitylation and degradation

**DOI:** 10.1111/acel.13367

**Published:** 2021-06-08

**Authors:** De‐bo Zou, Zongyou Mou, Wenliang Wu, Haichun Liu

**Affiliations:** ^1^ Department of Orthopaedics The First Affiliated Hospital of Shandong First Medical University Jinan, Shandong China; ^2^ Department of Orthopedics Dezhou People’s Hospital Dezhou, Shandong China; ^3^ Department of Orthopedics Qilu Hospital of Shandong University Jinan, Shandong China

**Keywords:** apoptosis, degradation, FOXO3a, osteoblasts, TRIM33

## Abstract

This study aimed to probe into the effect of TRIM33 on oxidative stress‐induced apoptosis of osteoblasts in osteoporosis and to probe into the underlying mechanism. The apoptosis of osteoblasts was induced by H_2_O_2_ treatment and tested by flow cytometry. A mouse osteoporosis model was conducted by ovariectomy (OVX). The function of TRIM33 was assessed by *in vitro* and *in vivo* experiments. The mechanism of TRIM33 was determined by immunoprecipitation, immunofluorescent staining and co‐transfection experiments. Here, we found that TRIM33 expression was lessened in the osteoblasts of patients with osteoporosis and was positively correlated with the bone mineral density of these patients. FOXO3a and TRIM33 were co‐localized in the osteoblasts nuclei. TRIM33 silence boosted FOXO3a degradation in normal osteoblasts, while TRIM33 overexpression restrained FOXO3a degradation in H_2_O_2_‐treated osteoblasts. The binding of TRIM33 to CBP and its overexpression restrained CBP‐mediated FOXO3a acetylation, thereby attenuating FOXO3a ubiquitylation. The H_2_O_2_‐induced apoptosis of osteoblasts was restrained by TRIM33 overexpression, while the FOXO3a knockdown reversed this trend. The *in vivo* experiments corroborated that TRIM33 overexpression attenuated the OVX‐driven impacts in mice. In general, our findings expounded that TRIM33 protected osteoblasts against oxidative stress‐induced apoptosis in osteoporosis and that the underlying mechanism was the restraint of FOXO3a ubiquitylation and degradation.

## Graphical abstract

The mechanism flow diagram to a better understanding of the pivotal role of TRIM33. In the presence of oxidative stress, the expression of TRIM33 in osteoblasts was lessened and the lessened TRIM33 further boosted the acetylation modification of FOXO3a by CBP, thereby boosting the ubiquitination and degradation of FOXO3a protein, and boosting the oxidative stress and apoptosis of osteoblasts and aggravating osteoporosis.

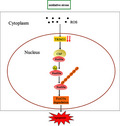

## 1 INTRODUCTION

Osteoporosis is systemic metabolic osteopathy; its topical characteristics are low bone mineral density (BMD), bone microstructure damage and increases the risk of fracture (Weitzmann & Pacifici, 2006). Although the incidence of osteoporosis is high and steadily raising (Sotorník, 2016), its pathogenesis remains largely unknown. The maintenance of bone mass depends not only on the absorption function of osteoclasts and the osteoblast function, but also on the difference between the production rate and apoptosis rate of osteoblasts and osteoclasts. Among them, osteoblasts play a crucial role in maintaining bone homeostasis and can regulate the mineralization of the cytoplasmic matrix and control bone remodeling and osteoclast differentiation (Shang, 2019). The apoptosis of osteoblasts promotes the occurrence and development of osteoporosis (Chen, 2017). Therefore, inhibiting the apoptosis of osteoblasts provides a new direction for the prevention and treatment of osteoporosis. A study has shown that oxidative stress plays a crucial role in the pathological process of all types of osteoporosis (Geng, 2019). During the onset of osteoporosis, the oxidative stress level of osteoblasts will significantly increase, indicating that the oxidative stress of osteoblasts plays a crucial role in pathological bone loss (Zhou & Liu, 2019; Cao, 2020). Oxidative stress not only inhibits osteogenic differentiation, but more importantly promotes the apoptosis of osteoblasts (Baek, 2010). Therefore, probing into the mechanism underlying the oxidative stress‐induced apoptosis of osteoblasts is important for understanding the pathogenesis of osteoporosis.

The tripartite motif (TRIM) family is a class of E3 ubiquitin ligases containing a ring domain, two B‐boxes and coiled‐coil domains (Venturini, 1999). Tripartite motif‐containing 33 (TRIM33/TIF1) is a subtype of the TRIM family and is bound up with various biological processes, such as mesendoderm differentiation (Xia, 2017), hematopoiesis (Ransom, 2004), granulomonopoiesis (Chretien, 2016), production and activation of macrophages (Gallouet, 2017), mitosis (Sedgwick, 2013) and tumorigenesis (Aucagne, 2011). Besides, TRIM33 plays a vital role in osteoblast differentiation. The enhanced TRIM33 expression facilitates osteoblast proliferation and differentiation, while the lessened TRIM33 expression has the opposite results (Guo, 2017). However, the effect of TRIM33 on oxidative stress‐induced apoptosis of osteoblasts in osteoporosis and the underlying mechanisms remain largely unknown.

Forkhead box O proteins (FOXOs), a family of transcription factors, are bound up with diverse biological processes, including cell proliferation, DNA damage repair and stress resistance (Calnan & Brunet, 2008). The functions of FOXOs are modulated by various post‐translational modifications, such as acetylation, phosphorylation and ubiquitylation (Zhu, 2014). FOXO3a (also known as FKHR‐L1) is a member of the FOXO family that is highly expressed in bones and osteoblasts (Jacobs, 2003; Ambrogini, 2010). This implies that FOXO3a plays an important role in bones and osteoblasts. A recent study reports that the raised transcriptional activity of FOXO3a is bound up with the role of parathyroid hormone in boosting DNA damage repair in osteoblasts and in ameliorating osteoporosis (Schnoke et al., 2009). The decreased FOXO3a expression boosts angiotensin II‐induced oxidative stress and DNA damage in osteoblasts (Li, 2014). The oxidative stress‐induced apoptosis of osteoblasts is raised in FOXO3a‐deleted mice, while the forced expression of FOXO3a in osteoblasts raises the BMD of mice (Ambrogini, 2010). This evidence expounds that the protective role of FOXO3a against the oxidative stress‐induced apoptosis of osteoblasts during osteoporosis development.

In our preliminary study, we applied mass spectrometry to screen for proteins that might interact with TRIM33 and found FOXO3a as a candidate protein, implying that TRIM33 might interact with FOXO3a. However, whether TRIM33 regulates the oxidative stress‐induced apoptosis of osteoblasts by modulating FOXO3a expression remains unclear. In this study, we aimed to address this question. The results expounded that TRIM33 protected osteoblasts from oxidative stress‐induced apoptosis in osteoporosis. The underlying mechanism was that TRIM33 restrained the CBP‐mediated FOXO3a acetylation, thereby blocking FOXO3a ubiquitylation and degradation.

## 2 METHODS AND MATERIALS

### 2.1 Clinical samples and primary human primary osteoblast isolation

Clinical bone tissue samples were gathered from 15 patients with osteoporosis confirmed by iDXA and X‐ray and 10 patients with osteoarthritis at our center. The bone tissues from patients with osteoarthritis were applied as the control for osteoporosis. The age of the patients ranged from 56 to 81 years old and the mean age of the patients was 65 years old. All patients had been informed of the purpose of this study and signed the consent. This study was approved by the Ethics Committee of Qilu Hospital of Shandong University.

Primary osteoblasts were separated from the trabecular bone of patients with osteoporosis and osteoarthritis given the previously described methods (Scimeca, 2017). Specifically, we washed the trabecular bone fragments with PBS and the bone fragments were incubated with 1 mg/ml Trypsin from porcine pancreas ≥60 /mg diluted in DPBS at room temperature. Then, we applied the 2.5 mg/ml Collagenase NB 4G Proved grade ≥0.18 U/mg diluted in DPBS with calcium and magnesium to digest the bone fragments. The supernatant was gathered and centrifuged to gather cell pellets, which were then cultured in the Dulbecco's modified Eagle's medium (DMEM; Gibco, Thermo Fisher) containing 15% fetal bovine serum (FBS; Thermo Fisher) and incubated at 37°C with 5% CO_2_ for nearly 4 weeks (cell fusion occurred). The alkaline phosphatase test was applied to characterize the osteoblasts.

### 2.2 Cell culture and H_2_O_2_ treatment

Primary human osteoblasts (HOB) were from PromoCell (Germany, Cat. No. C‐12720) and mouse preosteoblast cells (MC3 T3‐E1) were from ATCC (USA). HOB and MC3 T3‐E1 cells were put in the DMEM (Gibco, Thermo Fisher) and incubated at 37°C with 5% CO_2_. For inducing osteogenic differentiation, HOB and MC3 T3‐E1 cells were put in the DMEM with the addition of 10% FBS, 10 nM dexamethasone, 50 μg/ml ascorbic acid and 5 mM β‐glycerophosphate sodium for two weeks. The induced HOB and MC3 T3‐E1 cells were treated with 50 μM H_2_O_2_ for nearly 24 h.

### 2.3 Cell transfection

The small interfering RNA (siRNA) of FOXO3a (si‐FOXO3a), lentivirus (Lv) carrying siRNA of TRIM33 (Lv‐siTRIM33) and Lv expressing TRIM33 (Lv‐TRIM33) were synthesized by GenePharma. A scrambled control siRNA (sicontrol), Lv carrying sicontrol (Lv‐sicontrol) and Lv expressing green fluorescent protein (Lv‐GFP) were applied as the negative controls for si‐FOXO3a, Lv‐siTRIM33 and Lv‐TRIM33, respectively. These vectors were transfected into HOB and MC3 T3‐E1 cells using Lipofectamine 2000 (Invitrogen).

### 2.4 Quantitative real‐time PCR (qRT‐PCR)

Total RNAs were isolated from HOB and MC3 T3‐E1 cells using Trizol reagent (Invitrogen). After the quality and concentration were determined, 1000 ng RNA was applied to synthesize cDNA by the BeyoRT™ First Strand cDNA Synthesis Kit (Beyotime). The gathered cDNA was applied to conduct qRT‐PCR with BeyoFast™ SYBR Green qPCR Mix (Takara).

Gadd45a is an important gene in DNA repair (Wingert, 2016), and catalase is an important antioxidant enzyme (Glorieux & Calderon, 2017). The relative expressions of *Gadd45a* and *catalase* were tested using the 2^−ΔΔCT^ method. *GAPDH* was applied as the reference gene.

### 2.5 Western blot analysis

Total proteins were extracted from HOB and MC3 T3‐E1 cells, the primary osteoblasts isolated from the bone tissues of patients with osteoporosis and osteoarthritis or the osteoblasts isolated from the mouse bone marrow. The protein concentrations were tested using the BCA Protein Assay Kit (Beyotime). In total, 30 μg of protein from each sample was applied for SDS‐PAGE. After separation, the proteins were transferred into PVDF membranes (Roche, Basel, Switzerland). The PVDF membranes were blocked for nearly 1 h and then incubated with the primary antibodies anti‐FOXO3a (Abcam, 1/2500), anti‐TRIM33 (Abcam, 1/1000), anti‐Lamin B (Abcam, 1/2000), anti‐GAPDH (Abcam, 1/2500), anti‐CBP (Abcam, 1/1000), anti‐Gadd45a (Abcam, 1/1000), anti‐FOXO1 (Abcam, 1/2000), anti‐FOXO4 (Abcam, 1/2000) and anti‐catalase (Abcam, 1/2000) at 4°C for about 24 h. This was followed by the incubation with the corresponding secondary antibodies (Abcam, 1/2000) at room temperature for nearly 1 h. Protein bands were visualized using BeyoECL Moon (Beyotime). LaminB and GAPDH were applied as the references for nuclear protein and total protein, respectively.

### 2.6 Immunoprecipitation (IP) assays

For assessing the capacity of TRIM33 to bind to FOXO3a and CBP, the proteins isolated from HOB and MC3 T3‐E1 cells were incubated with 1 μg of antibodies against FOXO3a (Abcam, 1/2500), TRIM33 (Abcam, 1/1000) or CBP (Abcam, 1/1000) at 4 °C for nearly 24 h, followed by incubation with 100 μl protein A magnetic beads (Thermo Fisher) at room temperature for nearly 1 h. Then, the immunoprecipitated complex was analyzed by Western blot analysis using the corresponding antibodies.

For assessing FOXO3a acetylation, the proteins isolated from HOB and MC3 T3‐E1 cells were incubated with 1 μg of antibodies against acetylated lysine at 4 °C overnight, followed by the incubation with 100 μl protein A magnetic beads (Thermo Fisher) at room temperature for nearly 1 h. Then, the immunoprecipitated complex was assessed by Western blot using the antibody against FOXO3a.

For assessing FOXO3a ubiquitylation, HOB and MC3 T3‐E1 cells were co‐transfected with HA‐Ub and FLAG‐FOXO3a (or FOXO3a). Cell lysates were pre‐incubated with FLAG antibody at 4 °C for nearly 24 h, followed by the incubation with protein A magnetic beads at room temperature for nearly 1 h. Then, the immunoprecipitated complex was subjected to Western blot using an HA antibody. Besides, we conducted the TRIM33 RING domain with point mutations (C125A/C128A) to destroy its E3 ubiquitin ligase activity given the previously described methods (Xue, 2015).

### 2.7 Immunofluorescence staining

HOB and MC3 T3‐E1 cells were cultured on a coverslip. When cell confluence reached 95%–100%, the cells were washed with PBS thrice (10 min each time). After fixation in 4% formaldehyde (Aladdin) for nearly 20 min at room temperature, the cells were washed with PBS again. Then, the cells were permeabilized with 0.4% Triton X‐100 (Thermo Fisher) for nearly 1 h and blocked in BSA buffer for about 1 h at room temperature. Following this, the cells were incubated with the primary antibodies anti‐FOXO3a (Abcam, 5 µg/ml) and anti‐TRIM33 (Abcam, 1 µg/ml) for nearly 24 h at 4 °C and then incubated with FITC‐conjugated secondary antibody for about 1 h in the dark. The cells were then washed with PBS and stained with DAPI. The coverslips were sealed with glycerin and observed under a fluorescence microscope (Olympus, Japan).

### 2.8 Detection of ROS level, malondialdehyde (MDA) content, and superoxide dismutase (SOD) activity

The ROS level, MDA content and SOD activity in HOB and MC3 T3‐E1 cells were tested using the Reactive Oxygen Species Assay Kit (Beyotime), Lipid Peroxidation MDA Assay Kit (Beyotime) and Total Superoxide Dismutase Assay Kit with WST‐8 (Beyotime), given the manufacturers’ specifications.

### 2.9 Cell apoptosis detection

The apoptosis of HOB and MC3 T3‐E1 cells was tested using the Annexin V‐FITC Apoptosis Detection Kit (Beyotime). Single‐cell suspensions (1 × 10^5^) were incubated with 5 µl Annexin V‐FITC and 10 µl propidium iodide (PI) staining solution at room temperature in the dark. After incubation for nearly 15 min, 400 µl binding buffer was added to the cell suspensions. Then, the cells were passed through a flow cytometer (BD Biosciences). The apoptotic cells were assessed using FlowJo software.

### 2.10 Osteoporosis model establishment and adeno‐associated virus (AAV) injection

C57BL/6 J mice (8‐week‐old) were from the Shandong University Laboratory Animal Center and kept under the following standard conditions: temperature of 20–25°C, illumination cycle of 12 h light/dark and sufficient food and water. All animal experiments were approved by the Ethics Committee of Qilu Hospital of Shandong University. After 1 week of acclimation, the mice were applied to construct an osteoporosis model by ovariectomy (OVX) (Qiao, 2018). The mice were anesthetized using a mixture of ketamine (40 mg/kg) and Rompun^®^ (10 mg/kg) before the operation. The ovaries on both sides and the surrounding adipose tissues were removed from the OVX mice, while only the surrounding adipose tissues were removed from the sham mice.

Adeno‐associated virus 9 (AAV9) vectors carrying TRIM33 (AAV9‐TRIM33) or carrying GFP (AAV9‐GFP) were packaged by ViGene Biosciences. After the operation, a single dose of 1 × 10^11^ AAV9‐TRIM33 was injected into seven OVX mice via the tail vein and a single dose of 1 × 10^11^ AAV9‐GFP was injected into seven OVX mice and seven sham mice via the tail vein (Yang, 2019). Eight weeks after the operation, all the mice were sacrificed and their blood samples and bone marrow tissues were gathered for further analysis. Besides, the normal mice were injected with AAV9‐TRIM33 or AAV9‐GFP given the above method and the rest of the operation was as above.

### 2.11 Bone mineral density (BMD) and bone mineral content (BMC) assessment

The BMD and BMC of the mouse femur were assessed using the dual‐energy X‐ray absorptiometry method (Sanchez & Gilsanz, 2005). Following this, the ROI analysis was conducted.

### 2.12 Micro‐CT scanning

Micro‐CT scanning was conducted using Scanco vivaCT 40 (Scanco Medical AG). A region of interest in the distal femur was analyzed and defined as 1% of the total length proximal to the growth plate and extending 2 mm toward the diaphysis excluding the outer cortical bone. The X‐ray source was set at 60 kV and the femurs were scanned using a 6 μm pixel size. Parameters, such as bone volume fraction (BV/TV, %), trabecular number (Tb. N, number/mm), trabecular thickness (Tb. N, μm) and trabecular bone spacing (Tb. Sp, μm) were assessed based on the 3D images (Li, 2019).

### 2.13 Enzyme‐linked immunosorbent (ELISA) assay

Blood samples were gathered from the mice. The serum concentrations of amino‐terminal propeptide of type I collagen (PINP, a bone formation marker), tartrate‐resistant acid phosphatase type 5b (TRACP‐5b, a bone resorption marker) and estradiol (E2) were tested using the Mouse PINP ELISA Kit (MLBio), Mouse TRACP‐5b ELISA Kit (MLBio) and Mouse E2 ELISA Kit (Jining), respectively.

### 2.14 Statistical analysis

SPSS 22.0 software was employed to conduct data analysis. All data were expressed as mean ± standard deviation (SD). The difference between the two groups was assessed by the Student's t test, while the difference between more than two groups was evaluated by one‐way ANOVA and the Tukey–Kramer post hoc test. The Spearman's rank test was applied to conduct a correlation analysis. The *P*‐value of less than 0.05 indicated statistical significance.

## 3 RESULTS

### 3.1 TRIM33 expression in osteoblasts was positively correlated with the BMD of osteoporosis patients and the binding of TRIM33 to FOXO3a

**FIGURE 1:**
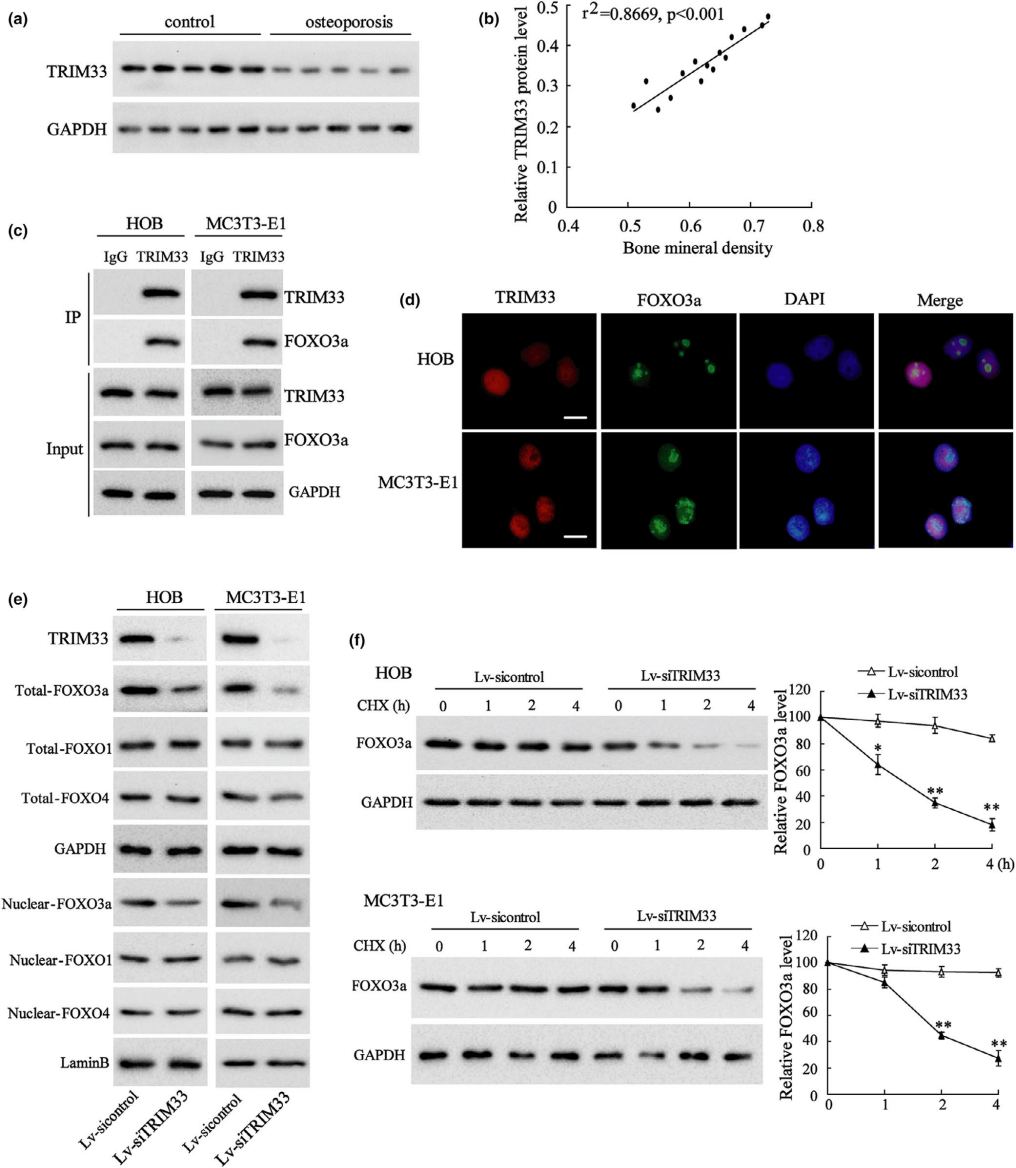
The expression of TRIM33 in clinical samples of patients with osteoporosis and TRIM33 bound to FOXO3a. (a) The protein level of TRIM33 in the osteoblasts from bone tissues from osteoporosis patients was tested by Western blot and five people are assigned to each group. (b) The correlation analysis between TRIM33 expression in osteoblasts and the bone mineral density (BMD) of osteoporosis patients (*n* = 15, r^2^=0.8669, *p*<0.001). (c) The binding capacity of TRIM33 to FOXO3a was tested by IP assay in HOB and MC3 T3‐E1 cells. (d) Representative images of immunofluorescence staining displaying the cellular localization of FOXO3a and TRIM33 in HOB and MC3 T3‐E1 cells. (e) HOB and MC3 T3‐E1 cells were transfected with Lv‐siTRIM33 or Lv‐sicontrol for 24 h. The protein levels of TRIM33, nuclear and total FOXO3a, FOXO1 and FOXO4 were tested by Western blot. (f) HOB and MC3 T3‐E1 cells were transfected with Lv‐siTRIM33 or Lv‐sicontrol for 24 h and then treated with CHX for indicated times (0, 1, 2 and 4 h). The protein level of FOXO3a was tested by Western blot. *p < 0.05, **p < 0.01 vs. Lv‐sicontrol group. The experiment was repeated three times

Western blot analysis confirmed a low expression of TRIM33 in the osteoblasts of patients with osteoporosis (Figure 1a). We then analyzed the correlation between TRIM33 expression and the BMD of patients with osteoporosis. As exhibited in Figure 1b, TRIM33 expression in osteoblasts was positively correlated with the BMD of patients with osteoporosis, implying a vital role of TRIM33 in osteoporosis. Mass spectrometry was conducted to screen for proteins that might interact with TRIM33. FOXO3a was identified as a candidate protein (Table S1). Moreover, the IP assay corroborated that TRIM33 was bound to FOXO3a in HOB and MC3 T3‐E1 cells (Figure 1c). Immunofluorescent staining analysis expounded that TRIM33 and FOXO3a were co‐localized in the nuclei of HOB and MC3 T3‐E1 cells (Figure 1d). We then assessed the effects of TRIM33 on the levels of FOXO3a and other FOXO proteins (FOXO1 and FOXO4). Western blot analysis corroborated that TRIM33 knockdown by Lv‐siTRIM33 lessened the levels of nuclear and total FOXO3a protein; while it had no significant effect on the levels of nuclear and total FOXO1 and FOXO4 proteins (Figure 1e). We then focused on the mechanism regulating the effect of TRIM33 on FOXO3a expression. As E3 ubiquitin ligases can mediate ubiquitylation and degradation and as TRIM33 is an E3 ubiquitin ligase, we assessed whether TRIM33 mediated FOXO3a degradation. Surprisingly, the results expounded that silencing TRIM33 contributed to FOXO3a degradation in the presence of cycloheximide (CHX, a protein synthesis inhibitor) (Figure 1f), hinting that TRIM33 did not regulate FOXO3a degradation via its E3 ubiquitin ligase function.

### 3.2 TRIM33 overexpression attenuated FOXO3a degradation in H_2_O_2_‐treated osteoblasts

**FIGURE 2:**
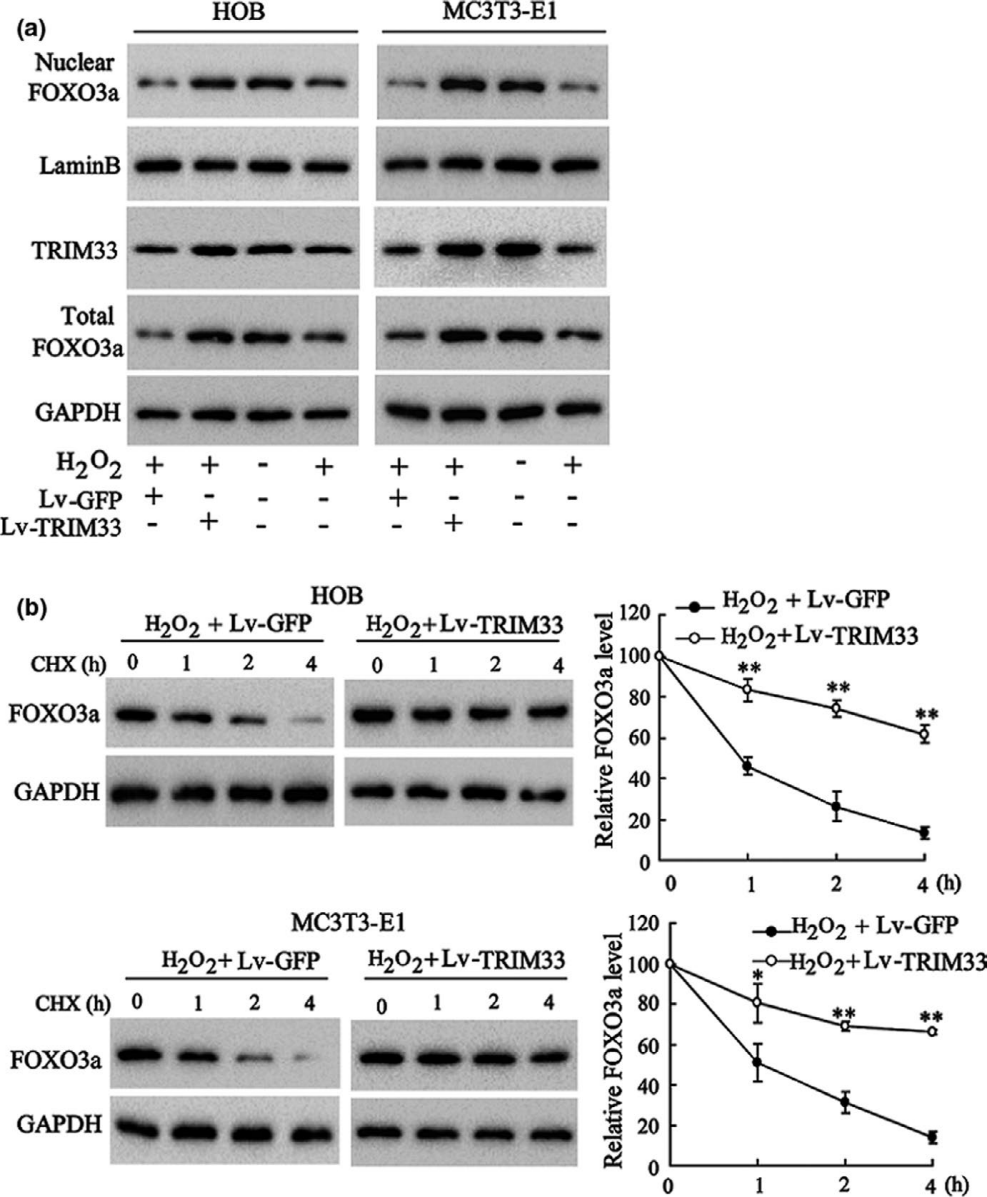
The effect of TRIM33 on FOXO3a expression in H_2_O_2_‐induced osteoblasts. (a) HOB and MC3 T3‐E1 cells were co‐transfected with Lv‐TRIM33 (or Lv‐GFP) for 24 h, followed by H_2_O_2_ (50 μM) treatment for another 24 h. The protein levels of TRIM33, nuclear and total FOXO3a were tested by Western blot. (b) HOB and MC3 T3‐E1 cells were transfected with Lv‐TRIM33 or Lv‐GFP for 24 h and then treated with H_2_O_2_ (50 μM) for another 24 h. Later, cells were treated with CHX for indicated times (0, 1, 2, and 4 h). The protein level of FOXO3a was tested by Western blot. ^*^ *p *< 0.05, ^**^ *p *< 0.01 vs. H_2_O_2_ + Lv‐GFP group. The experiment was repeated three times

We then probed into the effect of TRIM33 overexpression on FOXO3a degradation in osteoblasts under oxidative stress. H_2_O_2_ treatment lessened the protein levels of nuclear and total FOXO3a in HOB and MC3 T3‐E1 cells, while TRIM333 overexpression by Lv‐TRIM33 reversed these trends (Figure 2a). Also, TRIM33 overexpression restrained FOXO3a degradation in H_2_O_2_‐treated HOB and MC3 T3‐E1 cells (Figure 2b). Besides, we constructed the TRIM33 RING domain with point mutations (C125A/C128A), which destroys its E3 ubiquitin ligase activity (Xue, 2015). The results expounded that HOB cells transfected with Ad‐TRIM33‐CAmut reversed the lessening of nuclear FOXO3a and Total FOXO3a expression levels induced by H_2_O_2_ (Figure S1). These results corroborated that TRIM33 restrained FOXO3a degradation in H_2_O_2_‐treated osteoblasts.

### 3.3 TRIM33 overexpression restrained CBP‐mediated FOXO3a acetylation in H_2_O_2_‐treated osteoblasts

**FIGURE 3:**
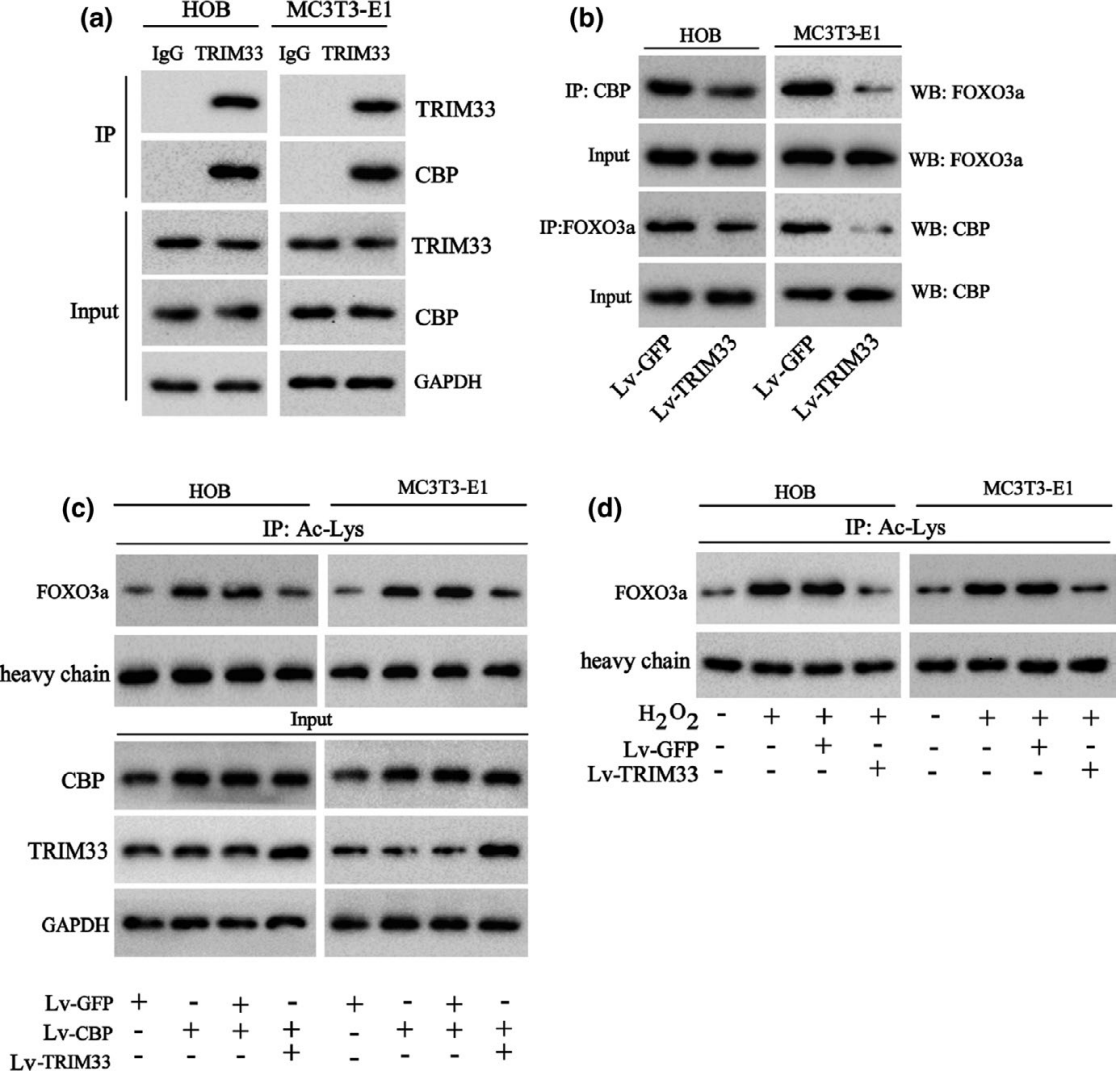
Effect of TRIM33 overexpression on the acetylation of FOXO3a. (a) The binding capacity of TRIM33 to CBP in HOB and MC3 T3‐E1 cells was tested by IP assay. (b) HOB and MC3 T3‐E1 cells were transfected with Lv‐TRIM33 or Lv‐GFP and then the binding capacity of TRIM33 to CBP in HOB and MC3 T3‐E1 cells was tested by IP assay. (c) HOB and MC3 T3‐E1 cells were transfected with Lv‐CBP and/or Lv‐TRIM33 . The acetylation of FOXO3a in cells was tested by IP assay and the protein levels of TRIM33 and CBP were tested by Western blot. (d) HOB and MC3 T3‐E1 cells were transfected with Lv‐TRIM33 or Lv‐GFP for 24 h and then ​the cells were treated with H_2_O_2_ (50 μM) for another 24 h. The acetylation of FOXO3a in cells was tested by IP assay. The experiment was repeated three times

As previous studies have corroborated that acetylation modification is an important mechanism affecting FOXO3a degradation and that the histone acetyltransferase CBP mediates FOXO3a acetylation (Senf, 2011; Wang, 2012, 2015), we further probed into whether TRIM33 regulated CBP‐mediated FOXO3a acetylation. Firstly, we probed into whether TRIM33 could bind to FOXO3a through IP assays. The results corroborated that TRIM33 bound to CBP in HOB and MC3 T3‐E1 cells (Figure 3a), hinting that TRIM33 formed a protein complex with CBP and FOXO3a. Secondly, we evaluated whether TRIM33 affected the capacity of FOXO3a to bind to CBP through IP assays. TRIM33 overexpression by Lv‐TRIM33 transfection restrained the capacity of FOXO3a to bind to CBP in HOB and MC3 T3‐E1 cells (Figure 3b). Thirdly, we probed into the effects of TRIM33 overexpression on CBP‐mediated FOXO3a acetylation in osteoblasts. The results of IP assays expounded that CBP overexpression boosted FOXO3a acetylation, while TRIM33 overexpression abolished this trend (Figure 3c). Finally, we probed into the effect of TRIM33 overexpression on FOXO3a acetylation in H_2_O_2_‐treated osteoblasts. As expected, H_2_O_2_ treatment induced FOXO3a acetylation in HOB and MC3 T3‐E1 cells, while TRIM33 overexpression abolished this trend (Figure 3d). These results hinted that TRIM33 attenuated CBP‐mediated FOXO3a acetylation in H_2_O_2_‐treated osteoblasts.

### 3.4 TRIM33 overexpression restrained FOXO3a ubiquitylation by blocking CBP‐mediated FOXO3a acetylation in H_2_O_2_‐treated osteoblasts

**FIGURE 4:**
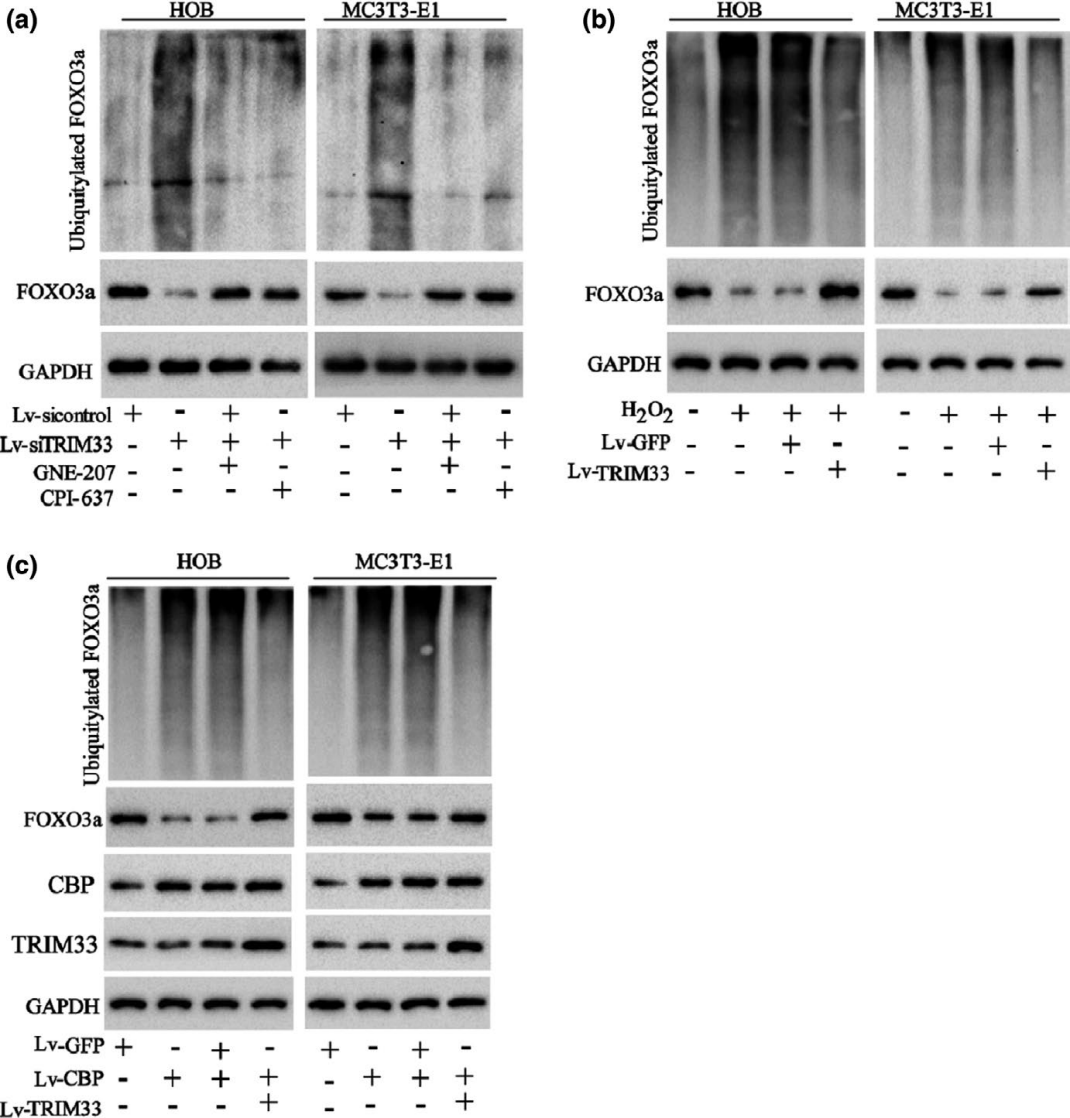
TRIM33 attenuated FOXO3a ubiquitylation by restraining its acetylation. (a) HOB and MC3 T3‐E1 cells were co‐transfected with HA‐Ub and Lv‐siTRIM33 (or Lv‐sicontrol) for 24 h and then treated with the CBP inhibitors GNE‐207 (5 nM) or CPI‐673 (50 nM). The ubiquitylation of FOXO3a was tested by IP assay. (b) HOB and MC3 T3‐E1 cells were co‐transfected with HA‐Ub and Lv‐TRIM33 (or Lv‐CBP) for 24 h and then treated with H_2_O_2_ (50 μM) for another 24 h. The ubiquitylation of FOXO3a was tested by IP assay. (c) HOB and MC3 T3‐E1 cells were transfected with Lv‐CBP and/or Lv‐TRIM33. The ubiquitylation of FOXO3a in cells was tested by IP assay, and the protein levels of TRIM33 and CBP were tested by Western blot. The experiment was repeated three times

FOXO3a acetylation can further affect FOXO3a ubiquitylation (Wang, 2012, 2015). As our results corroborated that TRIM33 restrained CBP‐mediated FOXO3a acetylation, we further probed into whether TRIM33 could affect FOXO3a ubiquitylation. As exhibited in Figure 4a, TRIM33 knockdown raised FOXO3a ubiquitylation in HOB and MC3 T3‐E1 cells, hinting that TRIM33 did not regulate FOXO3a expression via its E3 ubiquitin ligase function again. Besides, the treatment of CBP inhibitors (GNE‐207 or CPI‐637) hindered TRIM33 knockdown‐induced FOXO3a ubiquitylation in HOB and MC3 T3‐E1 cells, hinting that TRIM33 knockdown‐induced FOXO3a ubiquitylation was dependent on CBP‐mediated FOXO3a acetylation in osteoblasts. H_2_O_2_ treatment also boosted FOXO3a ubiquitylation in HOB and MC3 T3‐E1 cells, while TRIM33 overexpression reversed this effect (Figure 4b). Moreover, CBP overexpression by Lv‐CBP transfection facilitated FOXO3a ubiquitylation, while TRIM33 overexpression abolished this effect in HOB and MC3 T3‐E1 cells (Figure 4c). This corroborated that TRIM33 restrained FOXO3a ubiquitination by restraining CBP‐mediated FOXO3a acetylation in osteoblasts.

### 3.5 FOXO3a mediated the restraint of TRIM33 overexpression on oxidative stress and apoptosis of H_2_O_2_‐treated osteoblasts

**FIGURE 5:**
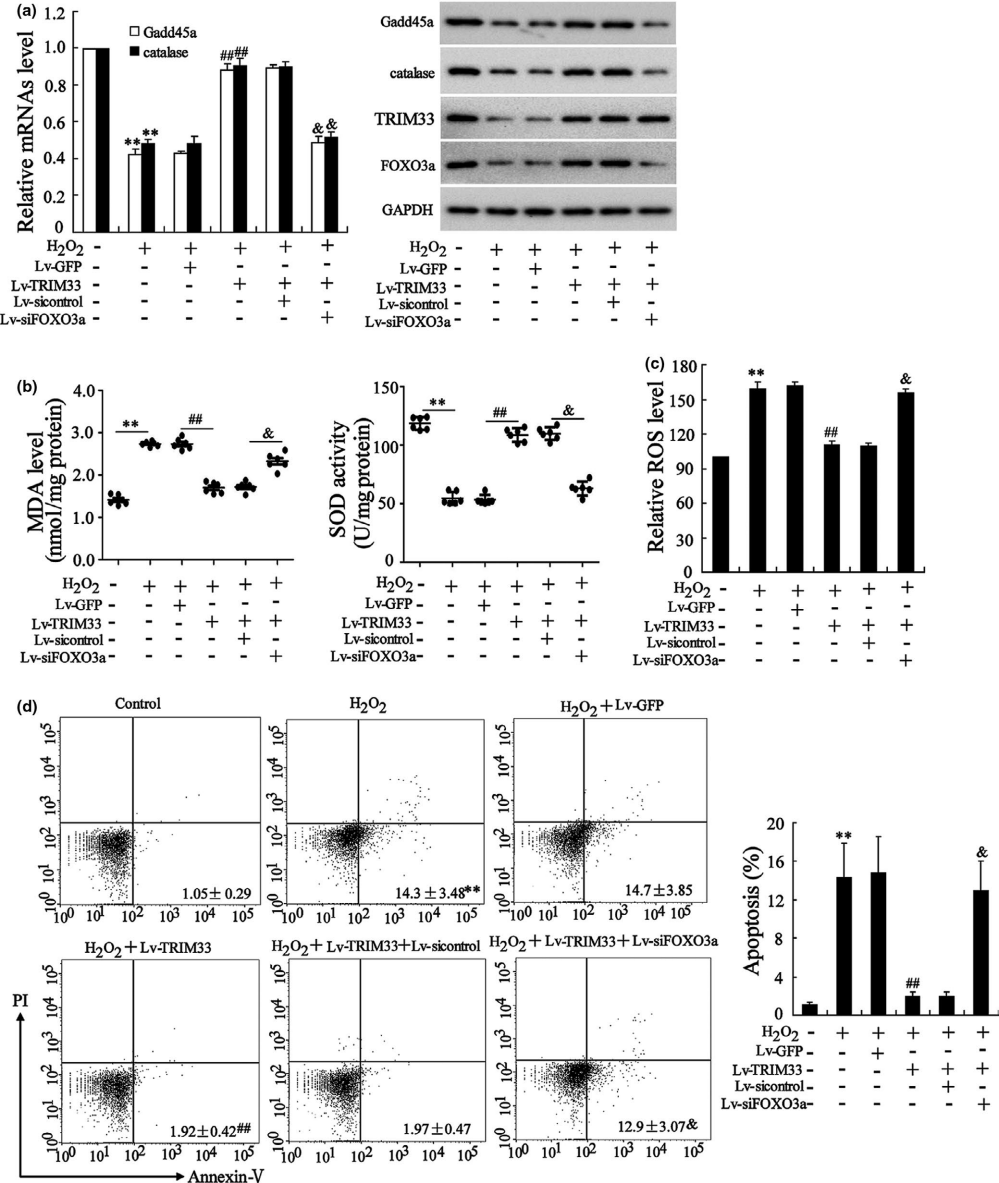
TRIM33 overexpression attenuated oxidative stress injury and apoptosis of osteoblasts through FOXO3a. HOB cells were transfected with Lv‐TRIM33, Lv‐siFOXO3a, or related negative controls (Lv‐GFP and Lv‐sicontrol) for 24 h and then treated with H_2_O_2_ (50 μM) for another 24 h. (a) The mRNA levels of Gadd45a and catalase were tested by qRT‐PCR and the protein levels of Gadd45a, catalase, TRIM33, and FOXO3a were tested by Western blot. (b) SOD activity and MDA level. (c) ROS level. (d) Cell apoptosis was tested by flow cytometry assay. ^**^ *p *< 0.01 vs. control group; ^##^ *p *< 0.01 vs. H_2_O_2_+Lv‐GFP group; ^&^ *p *< 0.05 vs. H_2_O_2_+Lv‐TRIM33+Lv‐sicontrol group. The experiment was repeated three times

Next, we probed into whether FOXO3a mediated the effect of TRIM33 on the oxidative stress‐induced apoptosis of H_2_O_2_‐treated osteoblasts. The results of qRT‐PCR and Western blot analysis expounded that TRIM33 overexpression augmented the mRNA and protein levels of Gadd45a (an important gene in DNA repair) and catalase (an important antioxidant enzyme) in H_2_O_2_‐treated HOB and MC3 T3‐E1 cells and that silencing FOXO3a reversed these trends (Figure 5a; Figure S2a), and the transfection efficiency was confirmed by Western blot (Figure S3). Besides, in the absence of H_2_O_2_ treatment, the overexpression of TRIM33 had no significant effect on the expressions of Gadd45a and Catalase, while knocking down FOXO3a lessened the expressions of Gadd45a and Catalase (Figure S3). The results also corroborated that the forced TRIM33 expression raised the activity of the antioxidant enzyme SOD and lessened the accumulation of MDA and ROS in H_2_O_2_‐treated HOB and MC3 T3‐E1 cells, while FOXO3a knockdown abolished these trends (Figure 5b, c; Figure S2b, c). Furthermore, the results of flow cytometry expounded that TRIM33 overexpression attenuated the apoptosis of H_2_O_2_‐treated HOB and MC3 T3‐E1 cells, while si‐FOXO3a transfection abolished these effects (Figure 5d; Figure S2d). Also, in the absence of H_2_O_2_ treatment, the overexpression of TRIM33 had no significant effect on cell apoptosis, while knocking down FOXO3a significantly promoted cell apoptosis (Figure S3). These findings corroborated that FOXO3a mediated the restraint of TRIM33 on the H_2_O_2_‐induced apoptosis of osteoblasts.

### 3.6 TRIM33 overexpression alleviated osteoporosis in OVX mice

**FIGURE 6:**
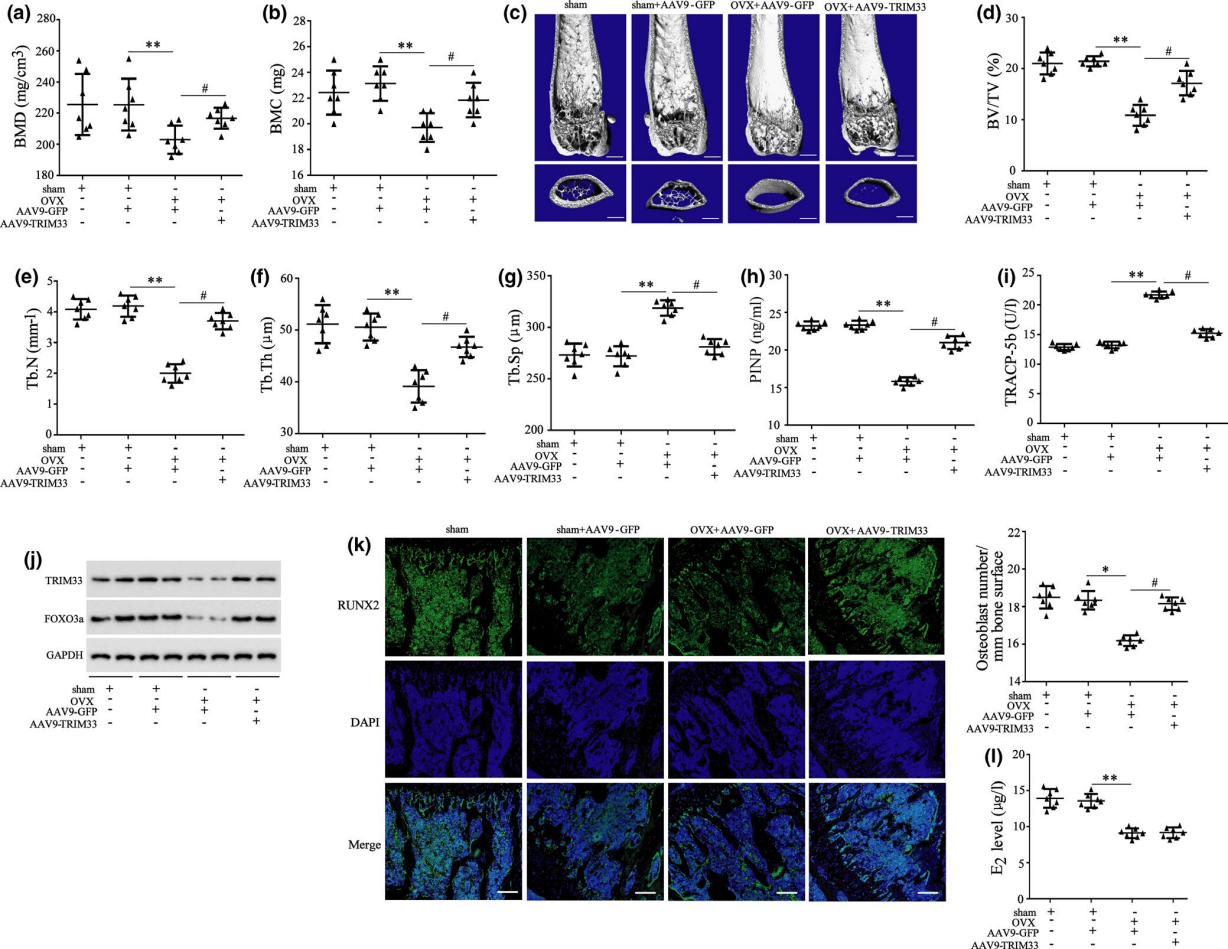
Effect of TRIM33 overexpression on osteoporosis of OVX mice. Mice were subjected to construct osteoporosis model by OVX operation and injected with AAV9‐TRIM33 or AAV9‐GFP by the tail vein, and seven mice were assigned to each group. Eight weeks after the model establishment, all the mice were sacrificed. (a, b) Then BMD and BMC of mouse femurs. (c) The image of Micro‐CT scanning of mouse femurs. Scale bars = 500 μm. (d–g) The BV/TV, trabecular number (Tb. N), trabecular thickness (Tb. Th), and trabecular spacing (Tb. Sp) of the femurs. (h) The concentration of PINP in serum. (i) The concentration of TRACP‐5b in serum. (j) The protein levels of TRIM33 and FOXO3a in the osteoblasts isolated from mouse bone marrow were tested by Western blot. (k) The detection of immunostained for RUNX2. (l) The detection of estradiol (E2) level in the serum samples. ^*^ *p *< 0.05, ^**^ *p *< 0.01 vs. Sham+AAV9‐GFP group; ^#^ *p *< 0.05 vs. OVX+AAV9‐GFP group. *n* = 7

Here, the methods of intra‐articulatory (I.A.) injected with adeno‐associated virus serotype 9 vector (AAV9) were applied to overexpress TRIM33, and the fluorescence microscopy was applied to test the expression of GFP in the hind legs and femur and corroborated that the osteoblast marker molecules Runx2 (red) and GFP (green) were co‐localized, hinting that the virus injected into the knee joint cavity could enter the osteoblasts (Figure S4). Then, we probed into the role of TRIM33 in osteoporosis *in vivo*. The results expounded that the mice in the OVX +AAV9‐TRIM33 group had higher BMD and BMC than those in the OVX +AAV9‐GFP group (Figure 6a, b). Moreover, in the normal mice, TRIM33 overexpressed had no significant influence on the BMD and BMC (Figure S5a, b). The results of micro‐CT scanning expounded that compared with the injection of AAV9‐GFP, the injection of AAV9‐TRIM33 raised the BV/TV, Tb. N, and Tb. Th of OVX mice, and lessened Tb. Sp of OVX mice (Figure 6c–g). Also, in the normal mice, TRIM33 overexpressed had no significant influence on the BV/TV, Tb. N, Tb. Th, and Tb. Sp (Figure S5e, f). The results of ELISA corroborated that TRIM33 overexpression elevated the serum concentration of PINP (a bone formation marker) and lessened the serum concentration of TRACP‐5b (a bone resorption marker) in OVX mice (Figure 6h, i). Furthermore, in the normal mice, TRIM33 overexpressed had no significant influence on the concentrations of PINP and TRACP‐5b (Figure S5g, h).

Moreover, the analysis of Western blot corroborated that TRIM33 overexpression elevated the levels of FOXO3a and TRIM33 proteins in the osteoblasts of OVX mice (Figure 6j). The detection of immunostained for RUNX2 expounded that TRIM33 overexpression reversed osteoblasts loss induced by OVX (Figure 6k). Furthermore, the detection of E2 level indicated that the serum E2 level was lessened in the OVX group, while TRIM33 overexpression had no significant effect on the serum E2 level (Figure 6l). These results hinted that TRIM33 overexpression alleviated osteoporosis in OVX mice.

## 4 DISCUSSION

The major novel findings of this study were exhibited: (1) TRIM33 expression in osteoblasts was positively correlated with the BMD of patients with osteoporosis; (2) TRIM33 raised the levels of FOXO3a protein and restrained its degradation; (3) TRIM33 restrained CBP‐mediated FOXO3a acetylation, thereby restraining FOXO3a ubiquitylation and degradation; (4) TRIM33 protected against the oxidative stress‐induced apoptosis of osteoblasts by raising FOXO3a expression; and (5) TRIM33 overexpression alleviated osteoporosis in OVX mice.

TRIM33 is bound up with various diseases, such as human cancers (Cai, 2019; He, 2018), idiopathic inflammatory myopathies (Kim, 2019) and colonic inflammation (Petit, 2019). It is also bound up with the regulation of osteoblast proliferation and differentiation (Qiao, 2018). However, the effect of TRIM33 on osteoporosis and the oxidative stress‐induced apoptosis of osteoblasts has not been reported so far. In this study, we applied the Full Bilateral Ovariectomy to establish the OVX model in female C57BL/6 mice, because oophorectomy has become the most widely used animal model of "osteoporosis", which has a high success rate, good reproducibility and high credibility. The OVX model of osteoporosis in mice not only leads to bone loss due to raised osteoclasts but also plays an important role in the apoptosis of osteoblasts (Zhang, 2020). Therefore, we chose the OVX model of osteoporosis. Here, we corroborated that TRIM33 expression was lessened in the osteoblasts of patients with osteoporosis and OVX mice and was positively correlated with the BMD of patients with osteoporosis. The functional verification experiment expounded that TRIM33 overexpression lessened H_2_O_2_‐induced oxidative stress and apoptosis of osteoblasts and raised the BMD and BMC of OVX mice. Thus, this is the first study to elucidate the effect of TRIM33 on osteoporosis and oxidative stress‐induced apoptosis of osteoblasts.

FOXO3a is an important transcription factor that can protect against the oxidative stress‐induced apoptosis of many types of cells, including neuronal cells and endothelial progenitor cells (Peng, 2015; Wang, 2015). Besides, FOXO3a restrains the oxidative stress‐induced apoptosis of osteoblasts. For instance, Ambrogini (2010) expounded that FOXO3a overexpression restrains the oxidative stress‐induced apoptosis of osteoblasts and raises the vertebral BMD of old mice. In this study, the IP assay verified that FOXO3a could bind to TRIM33. Immunofluorescent staining corroborated that FOXO3a and TRIM33 were co‐localized in osteoblast nuclei. The levels of nuclear and total FOXO3a protein were lessened by TRIM33 knockdown in normal osteoblasts and were raised by TRIM33 overexpression in H_2_O_2_‐treated osteoblasts. Hence, we concluded that FOXO3a might mediate the role of TRIM33 in regulating the oxidative stress‐induced apoptosis of osteoblasts. As expected, the functional recovery experiment corroborated that FOXO3a knockdown eliminated the effects of TRIM33 overexpression on the oxidative stress‐induced apoptosis of osteoblasts. Our findings hinted that FOXO3a mediated the role of TRIM33 in the oxidative stress‐induced apoptosis of osteoblasts.

TRIM33, an E3 ubiquitin ligase, can exert its E3 ubiquitin ligase function to ubiquitylate target proteins. For instance, TRIM33 binds to DHX33 and boosts its ubiquitylation at Lys63, resulting in the activation of the NLRP3 inflammasome (Weng, 2014); TRIM33 targets β‐catenin and mediates its ubiquitylation and degradation to restrain the proliferation of various types of cancer cells (Xue, 2015). Besides, TRIM33 has been proven to exert its function in a manner that does not depend on its E3 ligase activity. Xia (2017) corroborated that TRIM33 modulates the Wnt pathway in mouse embryonic stem cells, which is not bound up with its E3 ubiquitin ligase function. In this study, we corroborated that TRIM33 knockdown boosted FOXO3a degradation in normal osteoblasts, while TRIM33 overexpression restrained FOXO3a degradation in H_2_O_2_‐treated osteoblasts. Most importantly, TRIM33 knockdown contributed to FOXO3a ubiquitylation in normal osteoblasts. These results corroborated that the effect of TRIM33 on FOXO3a degradation was not dependent on its E3 ubiquitin ligase function. Besides, our data also found that the overexpression of TRIM33 reduced the level of bone resorption marker TRACP‐5b in the serum samples of OVX mice, suggesting that the overexpression of TRIM33 inhibited bone resorption in OVX mice and the possible mechanism would be explored in our future studies.

CBP an important histone acetyltransferase that can bind to FOXO3a and induce its acetylation (Senf, 2011; Matsuzaki, 2005). We corroborated that TRIM33 could also bind to FOXO3a and that TRIM33 overexpression hindered the capacity of CBP to bind to FOXO3a, hinting that TRIM33 might affect CBP‐mediated FOXO3a acetylation. The results of IP assays expounded that TRIM33 overexpression restrained CBP overexpression or H_2_O_2_‐induced FOXO3a acetylation in osteoblasts. As a novel insight that FOXO3a acetylation can affect the fate of FOXO3a has emerged recently (Wang, 2012, 2015), we further probed into whether TRIM33‐induced restraint of CBP‐mediated acetylation affected FOXO3a ubiquitylation. Interestingly, we corroborated that CBP inhibitors restrained TRIM33 knockdown‐induced FOXO3a ubiquitylation and that TRIM33 overexpression restrained CBP overexpression or H_2_O_2_‐induced FOXO3a ubiquitylation. This expounded that TRIM33 hindered CBP‐mediated FOXO3a acetylation and thus blocked FOXO3a ubiquitylation and degradation.

In summary, this study corroborated a protective role of TRIM33 against the oxidative stress‐induced apoptosis of osteoblasts in osteoporosis. The underlying mechanism was that TRIM33 bound to FOXO3a and restrained CBP‐mediated FOXO3a acetylation, subsequently restraining FOXO3a ubiquitylation and degradation. These findings might provide a novel insight into the effect of TRIM33 on osteoporosis and the underlying mechanism, which revealed a new therapeutic target for osteoporosis.

## CONFLICT OF INTEREST

The authors declare no conflict of interest.

## AUTHOR CONTRIBUTIONS

Zou DB and Liu HC designed the study and wrote the manuscript. Zou DB and Mou ZY did all experiments of this study. Zou DB and Wu WL collected the data and did the statistical analysis. All authors read and revised the manuscript.

## Supporting information

Fig S1

Fig S2

Fig S3

Fig S4

Fig S5

Table S1

## Data Availability

Data sharing not applicable to this article as no datasets were generated or analyzed during the current study.
