# Supplementary figures and images for: TRIM33 protects osteoblasts from oxidative stress‐induced apoptosis in osteoporosis by inhibiting FOXO3a ubiquitylation and degradation
Source: Aging Cell. 2021 Jun 8;20(7):e13367. doi: 10.1111/acel.13367 (PMC8282270; doi:10.1111/acel.13367)

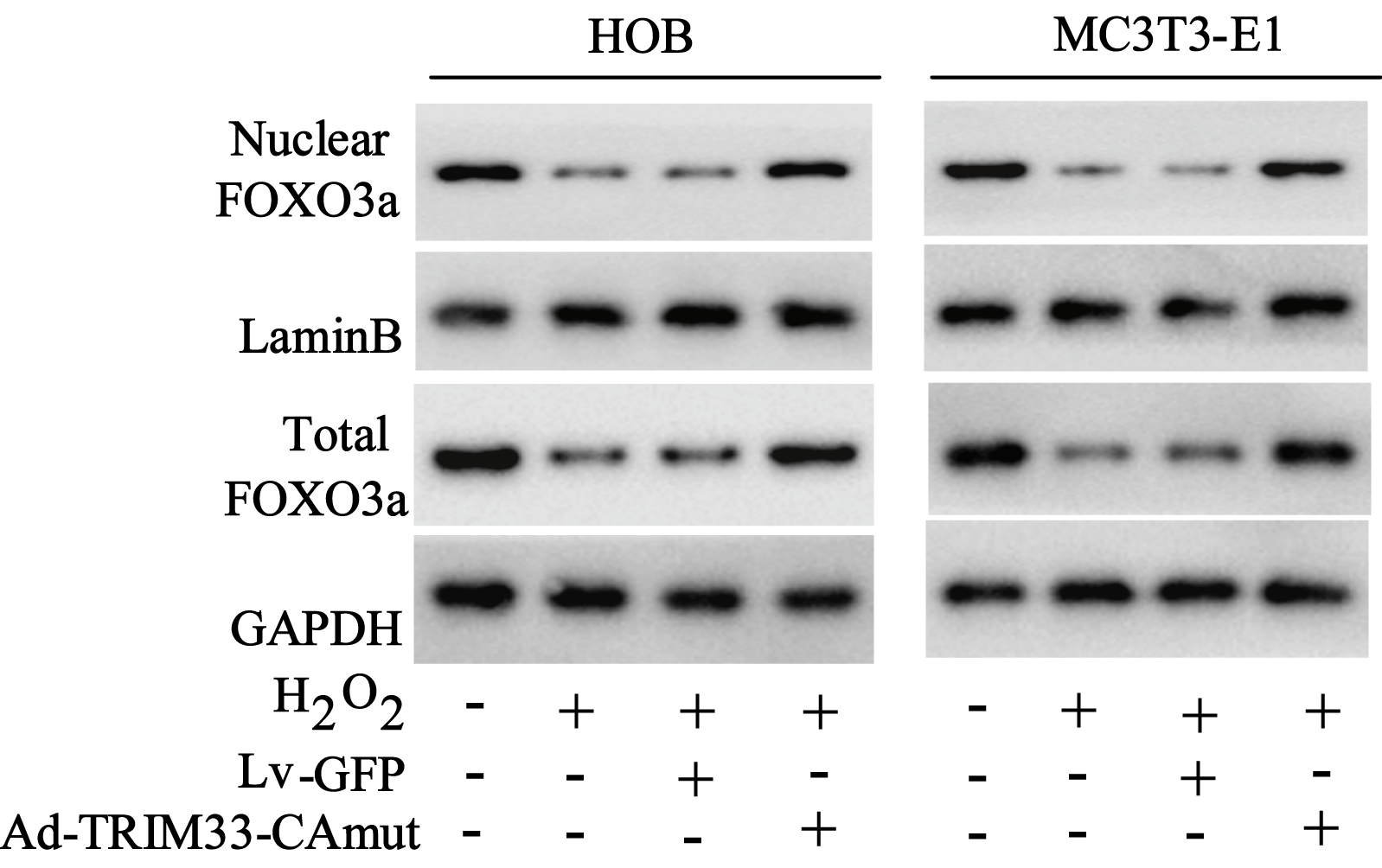

Supplement: Supplementary file 1 — Fig S1 [file ACEL-20-e13367-s004.tif]

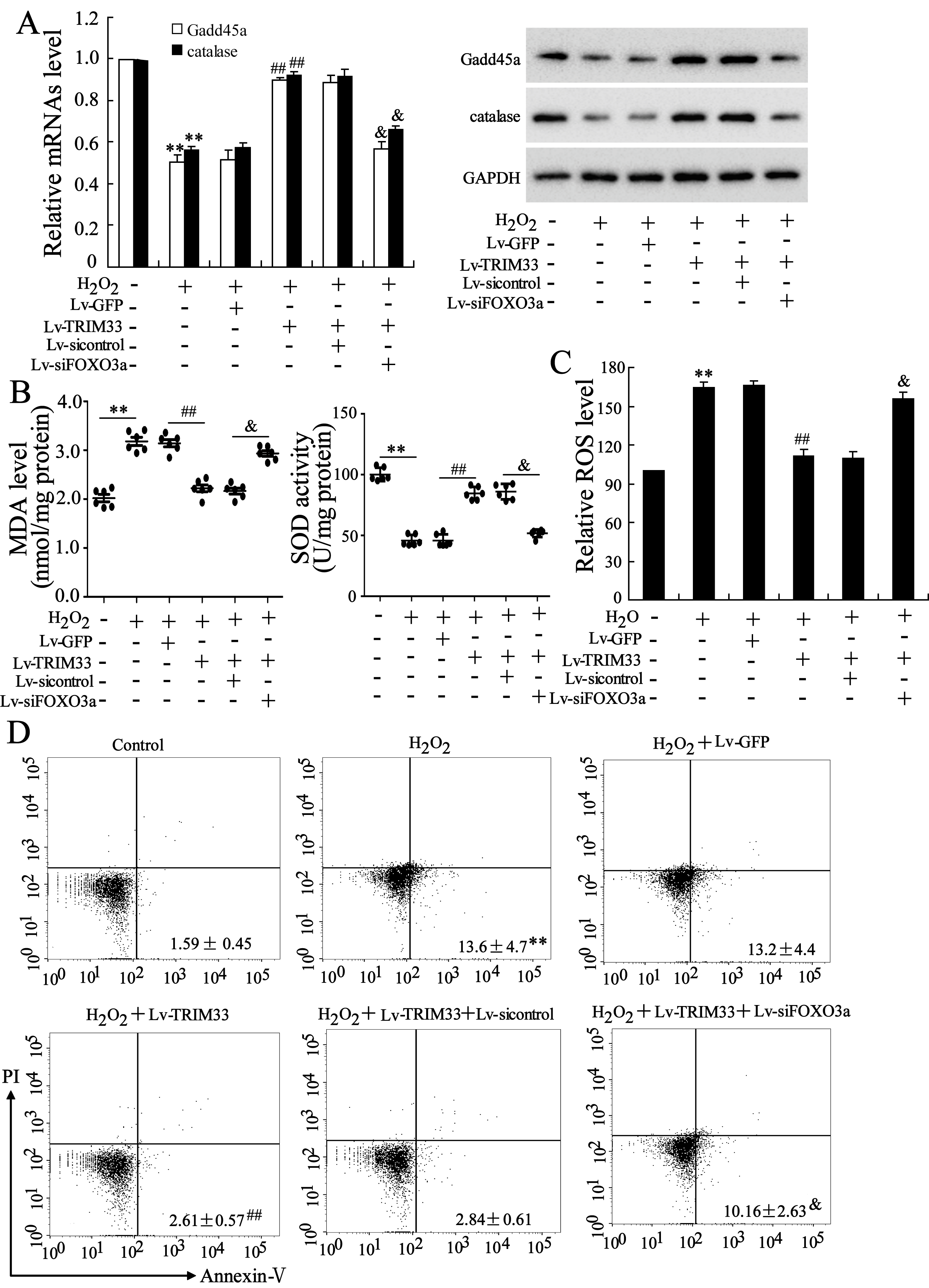

Supplement: Supplementary file 2 — Fig S2 [file ACEL-20-e13367-s002.tif]

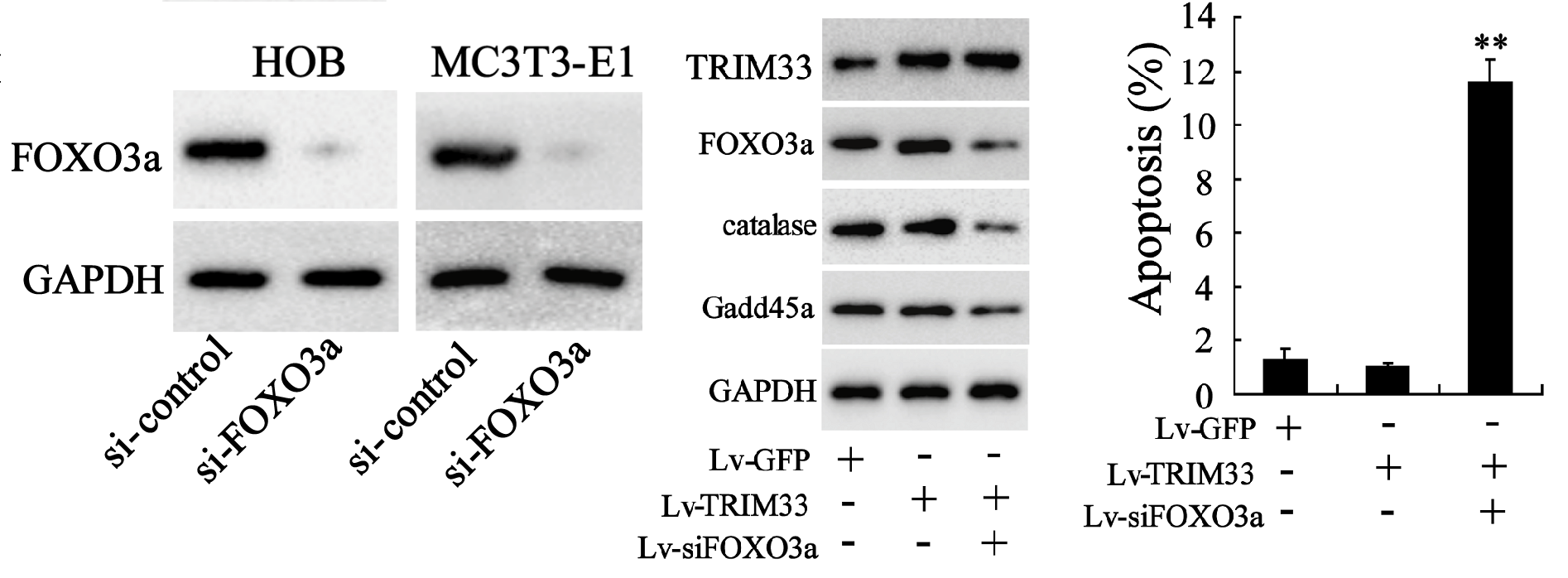

Supplement: Supplementary file 3 — Fig S3 [file ACEL-20-e13367-s003.tif]

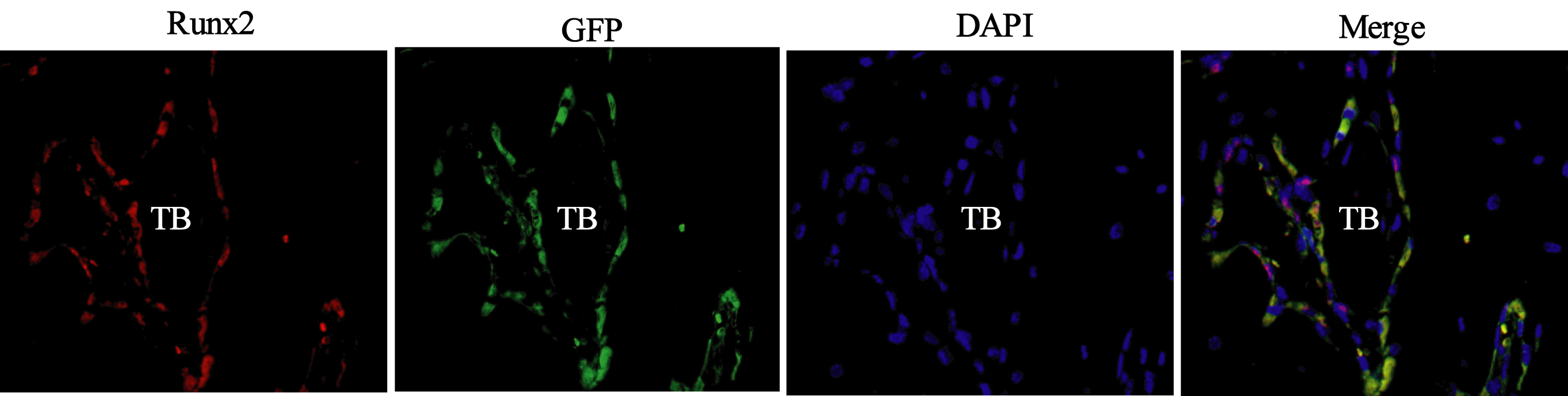

Supplement: Supplementary file 4 — Fig S4 [file ACEL-20-e13367-s001.tif]

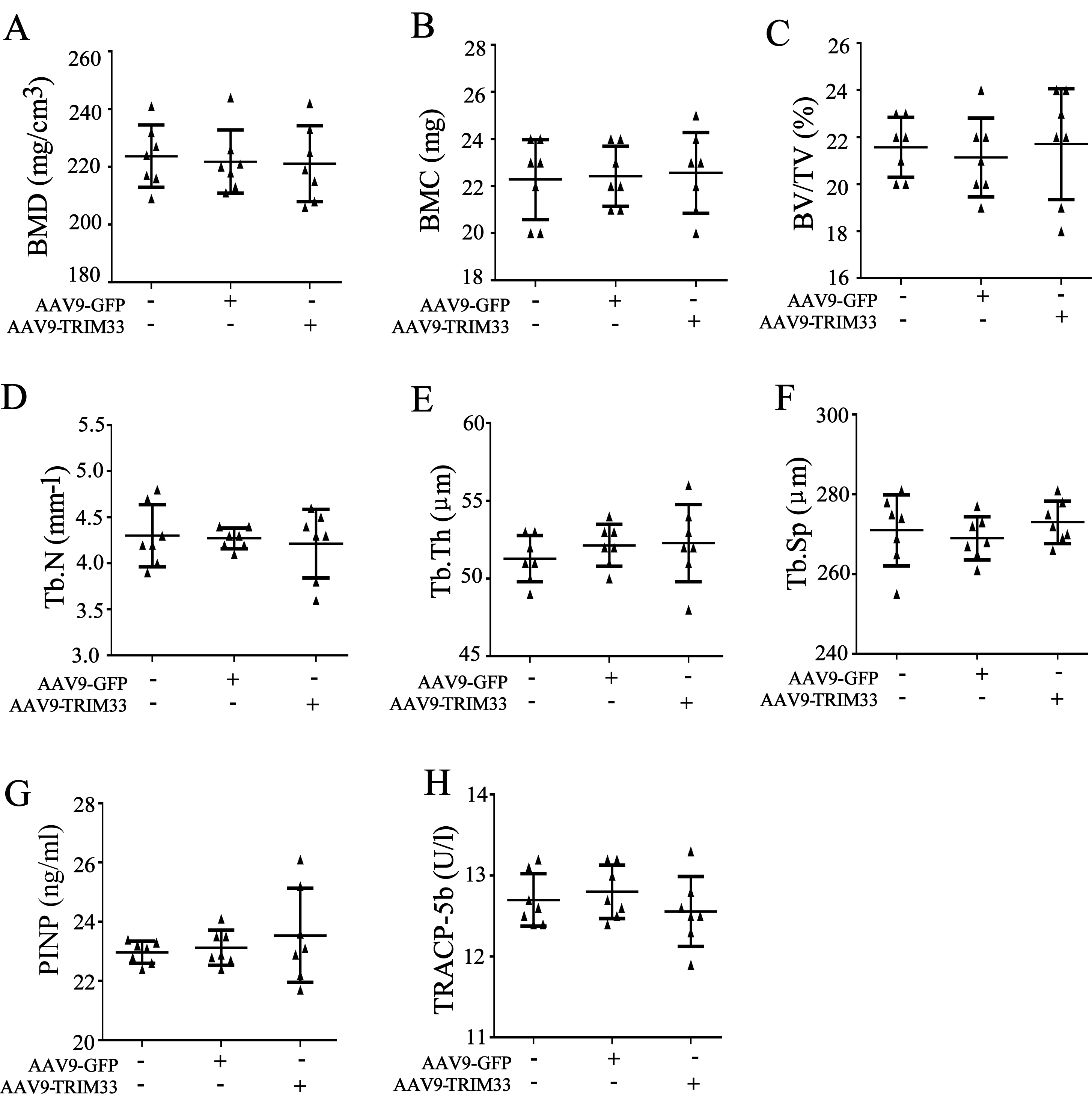

Supplement: Supplementary file 5 — Fig S5 [file ACEL-20-e13367-s006.tif]
