# Supplementary material for: TRIM33 protects osteoblasts from oxidative stress‐induced apoptosis in osteoporosis by inhibiting FOXO3a ubiquitylation and degradation
Source: Aging Cell. 2021 Jun 8;20(7):e13367. doi: 10.1111/acel.13367 (PMC8282270; doi:10.1111/acel.13367)
Supplement: Supplementary file 6 — Table S1 [file ACEL-20-e13367-s005.doc]

**Table 1** LC-MS/MS Analysis of TRIM33 Affinity-Purified Complexes

| **Gene** | **Protein** | **CT TSC^a^** | **TRIM33 TSC^b^** | **Ratio (b to a)** |
| --- | --- | --- | --- | --- |
| Hspa9 | Stress-70 protein, mitochondrial | - | + |  |
| Atp5a1 | ATP synthase subunit alpha | - | + |  |
| Foxo3a | Forkhead box O3 | - | + |  |
| EIF2S2 | Eukaryotic translation initiation factor 2 subunit 2 | - | + |  |
| Igkc | Immunoglobulin kappa constant (Fragment) | - | + |  |
| RPL3 | 60S ribosomal protein L3 | - | + |  |
| ACTB | Actin, cytoplasmic 1 | - | + |  |
| Rps16 | 40S ribosomal protein S16 | - | + |  |
| Iap | IgE-binding protein | + | + | 1025 |
| Snrpe | Small nuclear ribonucleoprotein E | + | + | 873 |
| ATF4 | Activating transcription factor 4 | + | + | 221 |
| CDH5 | Cadherin-5 | + | + | 85 |
| Txn | Thioredoxin | + | + | 24 |
| Gm15013 | 40S ribosomal protein | + | + | 8.2 |
| ZC3H12B | Probable ribonuclease ZC3H12B | + | + | 2.5 |
| Tpm4 | Tropomyosin 4 | + | + | 1.06 |
| ATRX | Transcriptional regulator ATRX | + | + | 0.62 |
| Tubb5 | Tubulin beta-5 chain | + | + | 0.42 |
| Sost | Sclerostin | + | + | 0.08 |
| FRAT1 | Frequently rearranged in advanced T-cell lymphomas 1 | + | + | 0.02 |

**Abbreviation**: CT, Control; TSC, total spectral counts. ^a^MC3T3-E1 cells infected with an empty lentivirus, not expressing TRIM33. ^b^MC3T3-E1 cells infected with a lentivirus expressing TRIM33.
